# Supplementary material for: The association of micro and macro worries with psychological distress in people living with chronic kidney disease during the COVID-19 pandemic
Source: PLoS One. 2024 Oct 22;19(10):e0309519. doi: 10.1371/journal.pone.0309519 (PMC11495632; doi:10.1371/journal.pone.0309519)
Supplement: S1 Text — (DOCX) [file pone.0309519.s001.docx]

**S1 Text. England (UK) COVID-19 lockdown and restrictions overview**

**England (UK) restrictions**. More detailed and further information can be found:

- <https://www.instituteforgovernment.org.uk/sites/default/files/timeline-lockdown-web.pdf>
- <https://www.instituteforgovernment.org.uk/sites/default/files/timeline-coronavirus-lockdown-december-2021.pdf>

**Timepoint 1 (August 2020 - November 2020)**

August Leisure centres such as indoor theatres, bowling alleys, and soft play centres reopened (following reopening of schools and non-essential shops (June), pubs, restaurants, and hairdressers (July)), local full lockdowns in pockets of the country.

September ‘Rule of six’ introduced, social gatherings both indoors and outdoors were limited to six people. Workers instructed to work from home and a 10pm curfew was imposed on hospitality venues.

October A new three-tier system was imposed, with different areas of England under different Tiers of restrictions (more information can be found at

<https://www.ageuk.org.uk/information-advice/coronavirus/coronavirus-guidance/local-lockdown-tiers/>)

November A second national lockdown came into force.

**Timepoint 2 (May 2021 – June 2021)**

May Limit of 30 people allowed to mix outdoors and ‘rule of six’ applied for indoor gatherings, indoor venues and sports stadiums reopened (following the introduction of the vaccine programme in December, the third national lockdown lifting in February, schools reopening and ‘rule of six’ outdoor gatherings introduced in March, non-essential indoor/outdoor retail and leisure venues, and outdoor hospitality venues opening in April)

**June** Acceleration of vaccination programme (offering a first vaccine to most adults, more details can be found here: <https://www.nao.org.uk/wp-content/uploads/2022/02/The-rollout-of-the-COVID-19-vaccination-programme-in-England.pdf>)
