# Supplementary material for: The association of micro and macro worries with psychological distress in people living with chronic kidney disease during the COVID-19 pandemic
Source: PLoS One. 2024 Oct 22;19(10):e0309519. doi: 10.1371/journal.pone.0309519 (PMC11495632; doi:10.1371/journal.pone.0309519)
Supplement: S2 Text — (DOCX) [file pone.0309519.s002.docx]

**S2 Text. Worry items revised from the WHO (2020) COVID-19 survey guidance**

Crises often involve fears and worries. Please let us know at the moment, how much do you worry about...

*7-point Likert scale from 1 (“do not worry at all”) to 7 (“worry a lot)*

1. Losing someone I love.
2. Health system being overloaded.
3. My own mental health.
4. My own physical health.
5. My loved ones’ health.
6. Restricted liberty of movement.
7. Losing holiday opportunities.
8. Economic recession in my country.
9. Restricted access to essential supplies.
10. Becoming unemployed.
11. Not being able to pay my bills.
12. Not be able to visit people who depend on me.
13. Having to defend a decision not to participate in a social event which my family or friends expect me to attend.
