# Supplementary material for: The association of micro and macro worries with psychological distress in people living with chronic kidney disease during the COVID-19 pandemic
Source: PLoS One. 2024 Oct 22;19(10):e0309519. doi: 10.1371/journal.pone.0309519 (PMC11495632; doi:10.1371/journal.pone.0309519)
Supplement: S1 Table — (DOCX) [file pone.0309519.s003.docx]

**S3 Table. Mean rating of worry items (including “worry about becoming unemployed”) for employed participants**

**TABLE S3** The mean rating of worry items for all employed participants at T1, and employed participants included in the multiple regression analyses at T1 and T2

|  | **Total (*N* = 528)** | | | **T1 (*N* = 245)** | | **T2 (*N* = 224)** | |
| --- | --- | --- | --- | --- | --- | --- | --- |
| **Worry** | **n** | **mean** | **± SD** | **mean** | **± SD** | **mean** | **± SD** |
| Losing a loved one | 238 | 4.8 | 1.9 | 4.9 | 1.9 | 4.0 | 1.8 |
| Healthcare system becoming overloaded | 239 | 5.0 | 1.6 | 5.0 | 1.6 | 4.5 | 1.5 |
| Mental health | 238 | 3.5 | 1.9 | 3.3 | 2.0 | 3.0 | 1.7 |
| Physical health | 239 | 4.5 | 1.9 | 4.3 | 1.9 | 3.9 | 1.8 |
| Loved one’s health | 238 | 5.2 | 1.7 | 5.2 | 1.8 | 4.4 | 1.6 |
| Restriction of movement | 237 | 3.6 | 1.9 | 3.5 | 2.0 | 3.2 | 1.9 |
| Losing holiday opportunities | 238 | 2.9 | 2.0 | 2.7 | 2.0 | 2.4 | 1.7 |
| Economic recession | 238 | 4.5 | 1.8 | 4.5 | 1.8 | 3.9 | 1.7 |
| Restricted access to essential supplies | 238 | 4.1 | 1.9 | 3.9 | 1.9 | 3.6 | 1.8 |
| Becoming unemployed | 236 | 3.6 | 2.3 | 3.5 | 2.3 | 2.8 | 2.0 |
| Not being able to pay bills | 238 | 3.7 | 2.3 | 3.6 | 2.2 | 2.7 | 1.8 |
| Not being able to visit dependents | 235 | 3.7 | 1.9 | 3.6 | 2.1 | 3.1 | 1.9 |
| Defending not socially participating | 237 | 2.7 | 2.1 | 2.6 | 2.0 | 2.1 | 1.5 |

Abbreviations: T1, timepoint 1; T2, timepoint 2.
