# Supplementary material for: The association of micro and macro worries with psychological distress in people living with chronic kidney disease during the COVID-19 pandemic
Source: PLoS One. 2024 Oct 22;19(10):e0309519. doi: 10.1371/journal.pone.0309519 (PMC11495632; doi:10.1371/journal.pone.0309519)
Supplement: S2 Table — Table A: Unadjusted model of association between worries and demographic factors at T1 and depression, anxiety, stress and health anxiety at T1; Table B: Unadjusted model of association between worries and demographic factors at T2 and depression, anxiety, stress and health anxiety at T2. (DOCX) [file pone.0309519.s004.docx]

**S4 Table. Unadjusted multiple regression models**

**TABLE A** Unadjusted model of association between worries and demographic factors at T1 and depression, anxiety, stress and health anxiety at T1 (*N* = 245)

|  | **Depression** | | | **Anxiety** | | | **Stress** | | | **SHAI** | | |
| --- | --- | --- | --- | --- | --- | --- | --- | --- | --- | --- | --- | --- |
| **Worry** | **(*N* = 240)** | | | **(*N* = 241)** | | | **(*N* = 244)** | | | **(*N* = 219)** | | |
|  | **β** | **CI** | **p** | **β** | **CI** | **p** | **β** | **CI** | **p** | **β** | **CI** | **p** |
| Losing a loved one | 0.07 | (-0.20, 0.58) | 0.380 | 0.01 | (-0.26, 0.30) | 0.893 | 0.03 | (-0.28, 0.41) | 0.668 | 0.21 | (0.24, 1.29) | 0.007 |
| Healthcare system becoming overloaded | 0.06 | (-0.20, 0.46) | 0.358 | 0.09 | (-0.11, 0.42) | 0.229 | 0.11 | (-0.03, 0.58) | 0.093 | 0.06 | (-0.31, 0.74) | 0.403 |
| Mental health | 0.44 | (0.59, 1.29) | <.001 | 0.30 | (0.18, 0.84) | 0.006 | 0.44 | (0.55, 1.25) | <.001 | 0.23 | (0.19, 1.35) | 0.007 |
| Physical health | 0.04 | (-0.25, 0.45) | 0.626 | 0.16 | (0.00, 0.55) | 0.042 | 0.06 | (-0.18, 0.46) | 0.435 | 0.37 | (0.80, 1.66) | <.001 |
| Loved one’s health | -0.04 | (-0.52, 0.31) | 0.656 | -0.03 | (-0.37, 0.25) | 0.742 | -0.01 | (-0.42, 0.40) | 0.940 | -0.10 | (-1.06, 0.29) | 0.232 |
| Restriction of movement | 0.10 | (-0.18, 0.65) | 0.249 | -0.11 | (-0.53, 0.12) | 0.247 | -0.04 | (-0.43, 0.23) | 0.534 | -0.03 | (-0.70, 0.44) | 0.645 |
| Losing holiday opportunities | -0.03 | (-0.37, 0.20) | 0.663 | -0.02 | (-0.23, 0.16) | 0.781 | -0.03 | (-0.33, 0.17) | 0.611 | -0.04 | (-0.61, 0.31) | 0.517 |
| Economic recession | -0.13 | (-0.64, 0.02) | 0.066 | -0.09 | (-0.49, 0.20) | 0.300 | -0.08 | (-0.54, 0.22) | 0.277 | -0.10 | (-0.94, 0.20) | 0.151 |
| Restricted access to essential supplies | 0.04 | (-0.23, 0.41) | 0.550 | 0.01 | (-0.26, 0.30) | 0.866 | 0.02 | (-0.24, 0.29) | 0.765 | 0.01 | (-0.40, 0.48) | 0.867 |
| Not being able to pay bills | 0.08 | (-0.14, 0.54) | 0.279 | 0.15 | (-0.01, 0.52) | 0.078 | 0.16 | (0.04, 0.59) | 0.031 | 0.07 | (-0.21, 0.70) | 0.312 |
| Not being able to visit dependents | -0.05 | (-0.37, 0.13) | 0.470 | 0.08 | (-0.10, 0.34) | 0.255 | 0.01 | (-0.19, 0.22) | 0.916 | 0.00 | (-0.40, 0.44) | 0.991 |
| Defending not socially participating | 0.00 | (-0.38, 0.41) | 0.967 | -0.02 | (-0.28, 0.22) | 0.772 | 0.05 | (-0.18, 0.40) | 0.453 | 0.05 | (-0.41, 0.81) | 0.537 |

β, standardised coefficient; CI, confidence interval; T1, timepoint 1.

Confidence intervals and p values are based on 1000 bootstrapped samples. Significance (*****) p<0.05

**TABLE B** Unadjusted model of association between worries and demographic factors at T2 and depression, anxiety, stress and health anxiety at T2 (N = 224)

|  | **Depression** | | | **Anxiety** | | | **Stress** | | | **SHAI** | | |
| --- | --- | --- | --- | --- | --- | --- | --- | --- | --- | --- | --- | --- |
| **Worry** | **(*N* = 221)** | | | **(*N* = 221)** | | | **(*N* = 216)** | | | **(*N* = 208)** | | |
|  | **β** | **CI** | **p** | **β** | **CI** | **p** | **β** | **CI** | **p** | **β** | **CI** | **p** |
| Losing a loved one | 0.10 | (-0.33, 0.72) | 0.379 | 0.10 | (-0.22, 0.58) | 0.403 | 0.00 | (-0.53, 0.52) | 0.990 | 0.04 | (-0.80, 1.04) | 0.688 |
| Healthcare system becoming overloaded | 0.03 | (-0.41, 0.58) | 0.694 | 0.06 | (-0.22, 0.47) | 0.445 | 0.09 | (-0.22, 0.72) | 0.281 | 0.03 | (-0.64, 1.00) | 0.690 |
| Mental health | 0.38 | (0.43, 1.51) | 0.002 | 0.24 | (0.06, 0.81) | 0.024 | 0.34 | (0.33, 1.32) | <.001 | 0.18 | (-0.08, 1.53) | 0.064 |
| Physical health | 0.09 | (-0.23, 0.61) | 0.346 | 0.13 | (-0.06, 0.50) | 0.192 | 0.00 | (-0.44, 0.38) | 0.980 | 0.22 | (-0.06, 1.74) | 0.041 |
| Loved one’s health | -0.10 | (-0.91, 0.42) | 0.371 | 0.01 | (-0.39, 0.50) | 0.893 | 0.08 | (-0.32, 0.83) | 0.439 | -0.01 | (-1.02, 1.03) | 0.917 |
| Restriction of movement | -0.03 | (-0.53, 0.42) | 0.757 | -0.17 | (-0.67, 0.03) | 0.057 | -0.06 | (-0.59, 0.33) | 0.496 | -0.08 | (-0.95, 0.31) | 0.393 |
| Losing holiday opportunities | -0.05 | (-0.56, 0.32) | 0.561 | 0.04 | (-0.22, 0.39) | 0.576 | -0.01 | (-0.46, 0.44) | 0.925 | 0.02 | (-0.68, 0.99) | 0.795 |
| Economic recession | 0.07 | (-0.25, 0.52) | 0.442 | 0.05 | (-0.18, 0.38) | 0.530 | 0.02 | (-0.36, 0.44) | 0.795 | 0.00 | (-0.65, 0.60) | 0.944 |
| Restricted access to essential supplies | -0.05 | (-0.62, 0.35) | 0.573 | -0.04 | (-0.44, 0.29) | 0.701 | 0.04 | (-0.35, 0.56) | 0.688 | -0.01 | (-0.80, 0.77) | 0.924 |
| Not being able to pay bills | 0.05 | (-0.37, 0.67) | 0.643 | 0.03 | (-0.31, 0.43) | 0.721 | 0.08 | (-0.34, 0.82) | 0.452 | 0.01 | (-0.61, 0.71) | 0.949 |
| Not being able to visit dependents | -0.02 | (-0.39, 0.32) | 0.836 | 0.05 | (-0.21, 0.37) | 0.606 | 0.07 | (-0.21, 0.53) | 0.408 | -0.05 | (-0.69, 0.38) | 0.528 |
| Defending not socially participating | 0.05 | (-0.38, 0.59) | 0.524 | 0.17 | (-0.03, 0.78) | 0.108 | 0.01 | (-0.46, 0.45) | 0.913 | 0.10 | (-0.31, 1.15) | 0.231 |

β, standardised coefficient; CI, confidence interval; T2, timepoint 2.

Confidence intervals are 95% bias corrected and accelerated. Confidence intervals and p values are based on 1000 bootstrapped samples. Significance (*****) p<0.05
