# Supplementary material for: The association of micro and macro worries with psychological distress in people living with chronic kidney disease during the COVID-19 pandemic
Source: PLoS One. 2024 Oct 22;19(10):e0309519. doi: 10.1371/journal.pone.0309519 (PMC11495632; doi:10.1371/journal.pone.0309519)
Supplement: S3 Table — (DOCX) [file pone.0309519.s005.docx]

**S5 Table. Descriptive statistics of demographics for participants not included in the multiple regression analyses**

**TABLE S5** Demographic characteristics of all participants who were not included in the multiple regression analyses at T1

| **Demographic** | **Total (*N* = 283)** | |
| --- | --- | --- |
| **Age** | 60.0 | ± 12.4 |
| **IMDD** | 6.4 | ± 2.6 |
| **Type of Kidney Problem** |  |  |
| NDCKD | 160 | 56.5% |
| eGFR, ml/min/1.73m2 | 34.6 | ± 20.9 |
| KTR | 123 | 43.5% |
| **Gender** |  |  |
| Female | 123 | 43.5% |
| Male | 159 | 56.2% |
| **Ethnicity** |  |  |
| White British | 262 | 92.6% |
| Other White | 5 | 1.8% |
| South Asian | 4 | 1.4% |
| Other Ethnicity | 12 | 4.2% |
| **Education** |  |  |
| None | 16 | 5.7% |
| High school | 79 | 27.9% |
| College | 79 | 27.9% |
| Trade qualification | 27 | 9.5% |
| University | 80 | 28.3% |
| **Employment Status** |  |  |
| Employed | 109 | 38.5% |
| Self-employed | 31 | 11.0% |
| Retired | 124 | 43.8% |
| Unemployed | 8 | 2.8% |
| Carer / Homemaker | 7 | 2.5% |
| Student | 2 | 0.7% |
| Other | 7 | 2.5% |
| **Comorbidities** |  |  |
| Hypertension | 214 | 75.6% |
| Diabetes type II | 55 | 19.4% |
| Mental health issues | 60 | 21.2% |

Abbreviations: IMDD, index of multiple deprivation decile; eGFR, estimated glomerular filtration rate; NDCKD, non-dialysis chronic kidney disease; KTR, kidney transplant recipient. Data are presented as mean ± SD, or *n* (%)
